# Direct visualization of cGMP microdomains in adult cardiomyocytes

**DOI:** 10.1186/2050-6511-16-S1-A91

**Published:** 2015-09-02

**Authors:** Hariharan Subramanian, Alexander Froese, Daniela Hübscher, Konrad R Götz, Viacheslav O Nikolaev

**Affiliations:** 1 Institute for Experimental Cardiovascular Research, University Medical Center Hamburg-Eppendorf (UKE), Hamburg, Germany; 2 Heart Research Center Göttingen, University of Göttingen Medical Center (UMG), Göttingen, Germany

## Background

cGMP is an important second messenger which regulates cardiac function and might protect the heart from hypertrophy and failure by acting in distinct subcellular microdomains. However, direct visualization of cGMP in such microdomains of adult cardiomyocytes has been challenging due to low sensitivity of the available real-time imaging techniques.

## Methods

We used a highly sensitive cytosolic Förster resonance energy transfer (FRET)-based biosensor, red cGES-DE5 (affinity for cGMP ~40 nM) to generate its targeted versions and transgenic mice expressing them in adult mouse myocardium to monitor cGMP in freshly isolated adult ventricular cardiomyocytes.

## Results

Cardiomyocytes were isolated from the transgenic mice expressing the cytosolic red cGES-DE5 sensor [1]. Using the previously established scanning ion conductance microscopy (SICM)/FRET method [2], local application of Atrial Natriuretic Peptide (ANP) at T-tubules and crests revealed the exclusive localization of guanylyl cyclase A (GC-A) in T-tubules. In contrast, guanylyl cyclase B (GC-B), the receptor for the C-type Natriuretic Peptide (CNP), was more evenly distributed across the membrane. Further, two differentially targeted cGMP sensors were generated. One of them was targeted to the caveolin-rich membrane microdomains via a palmitoylation and myristoylation motif (pmDE5) and another one to the sarcoplasmic reticulum by the fusion with phospholamban (DE5-PLN). FRET measurements in cardiomyocytes from these transgenic mice revealed close association of the GC-A with the caveolin-rich membrane microdomains, as well as a CNP, but not ANP-dependent regulation of cGMP levels at the sarcoplasmic reticulum.

## Conclusion

FRET and SICM/FRET approaches with freshly isolated cardiomyocytes represents a powerful way of dissecting local cGMP signals in distinct cardiac microdomains. It reveals distinct cGMP microdomains associated with various GCs.
